# The Synergy of Arbuscular Mycorrhizal Fungi and Exogenous Abscisic Acid Benefits *Robinia pseudoacacia* L. Growth through Altering the Distribution of Zn and Endogenous Abscisic Acid

**DOI:** 10.3390/jof7080671

**Published:** 2021-08-19

**Authors:** Xiao Lou, Xiangyu Zhang, Yu Zhang, Ming Tang

**Affiliations:** 1 College of Forestry, Northwest A&F University, Yangling 712100, China; louxiao2021@nwsuaf.edu.cn (X.L.); zhangxy0588@outlook.com (X.Z.); yuzhang20210720@163.com (Y.Z.); 2 State Key Laboratory for Conservation and Utilization of Subtropical Agro-Bioresources, Lingnan Guangdong Laboratory of Modern Agriculture, Guangdong Key Laboratory for Innovative Development and Utilization of Forest Plant Germplasm, College of Forestry and Landscape Architecture, South China Agricultural University, Guangzhou 510642, China

**Keywords:** arbuscular mycorrhizal fungus, excess Zn stress, abscisic acid, proline metabolism, antioxidant response

## Abstract

The simultaneous effects of arbuscular mycorrhizal (AM) fungi and abscisic acid (ABA) on the tolerance of plants to heavy metal (HM) remain unclear. A pot experiment was carried out to clarify the effects of simultaneous applications of AM fungi and ABA on plant growth, Zn accumulation, endogenous ABA contents, proline metabolism, and the oxidative injury of black locust (*Robinia pseudoacacia* L.) exposed to excess Zn stress. The results suggested that exogenously applied ABA positively enhanced AM colonization, and that the growth of plants only with AM fungi was improved by ABA application. Under Zn stress, AM inoculation and ABA application increased the ABA content in the root/leaf (increased by 48–172% and 92%, respectively) and Zn content in the root/shoot (increased by 63–152% and 61%, respectively) in AM plants, but no similar trends were observed in NM plants. Additionally, exogenous ABA addition increased the proline contents of NM roots concomitantly with the activities of the related synthases, whereas it reduced the proline contents and the activity of Δ^1^-pyrroline-5-carboxylate synthetase in AM roots. Under Zn stress, AM inoculation and ABA application decreased H_2_O_2_ contents and the production rate of O_2_, to varying degrees. Furthermore, in the roots exposed to Zn stress, AM inoculation augmented the activities of SOD, CAT, POD and APX, and exogenously applied ABA increased the activities of SOD and POD. Overall, AM inoculation combined with ABA application might be beneficial to the survival of black locust under Zn stress by improving AM symbiosis, inhibiting the transport of Zn from the roots to the shoots, increasing the distribution of ABA in roots, and stimulating antioxidant defense systems.

## 1. Introduction

With growing industrialization and urban expansion, China faces significant challenges regarding soils severely contaminated with heavy metals (HMs) [1]. The total amount of soils contaminated with zinc (a heavy metal) pollutants accounts for 0.9% of the total number of soils samples in China (Ministry of Environmental Ministry of Land and Resources, 2014). Zinc (Zn) is one of the microelements and participates in a variety of biological processes in plants and animals [2]. However, high Zn concentrations can be toxic to plants [2], and severely threaten human health by damaging the nervous system [3]. Consequently, plants have evolved various mechanisms to circumvent the adverse effects caused by excessive concentrations of metals: restricting metal transport to the aboveground parts [4] and decreasing heavy metal accumulation [5]. In addition, phytohormones such as cytokinins, abscisic acid, and salicylic acid (SA) are indicated to promote plant tolerance to heavy metal stress by increasing plant biomass and regulating the transport of HMs [6,7].

Abscisic acid (ABA), a vital plant stress hormone, has a pivotal function on plant responses to various abiotic stresses, such as heavy metals [8], salinity [9], and drought [10]. Previously, it was shown that heavy metal stress can affect endogenous ABA concentrations [11]. Increased endogenous ABA contents have been reported in *Populus × canescens* after exposure to heavy metal stress, which may alleviate heavy-metal-induced toxicity [8]. Previous research has also shown that ABA can stimulate antioxidant mechanisms that contribute to increased heavy metal tolerance [12]. Additionally, exogenous ABA application reduces heavy metal translocation from the roots to the aboveground parts of plants by decreasing transpiration and alleviating heavy metal toxicity by priming plant defenses [13]. Therefore, ABA-rich plants or plants with an exogenous ABA application have an increased potential for HM stress tolerance.

Arbuscular mycorrhizal (AM) fungi can establish mutual endosymbiosis with most terrestrial plants [14]. They are an important component of soil microorganisms and are abundant in soils, including soils contaminated with heavy metals [15]. AM symbiosis can exert protective effects on host plants under high soil heavy metal concentrations via mediating interactions between heavy metals and plant roots [16,17]. The inoculation with AM fungi is also considered to increase the absorption of nutrients (such as phosphorus and nitrogen) [18] and alter the distribution of heavy metals in different tissues [19] to promote the adaptation of host plants in heavy metal stressful conditions. Furthermore, AM fungi can ameliorate the oxidative damage induced by heavy metal stress through the induction of acquired systemic tolerance [19]. Therefore, AM plants have an increased potential for ameliorating heavy metal toxicity and improved tolerance. Several studies have shown that ABA also contributes to the regulation of the formation and development of AM symbiosis [20,21]. However, there are few studies which address the different mechanisms of mycorrhizal and non-mycorrhizal plants exposed to Zn stress in response to exogenous ABA application.

In this study, we used seedlings of black locust that were exposed to Zn stress, in combination with AM inoculation and exogenous ABA application, to explore the different responses to exogenous ABA between non-mycorrhizal and mycorrhizal plants exposure to Zn stress. The purposes of this study were as follows: (1) to examine whether AM inoculation and/or exogenous ABA application alleviates Zn toxicity in black locust, and (2) to elucidate the different mechanisms by which mycorrhizal and non-mycorrhizal black locusts exposed to Zn stress respond to exogenous ABA. For these purposes, we analyzed plant growth, AM symbiosis, ABA accumulation, Zn levels, proline response, and the antioxidant system. Insights into these responses to AM fungi and/or exogenous ABA under Zn stress will provide a new perspective for the remediation of Zn-contaminated soils by woody plants.

## 2. Materials and Methods

### 2.1. AM Fungi Inoculum Preparation

*Rhizophagus irregularis* (BGC BJ109), the AM fungi used in this study, was obtained from Beijing Academy of Agriculture and Forestry Science (Beijing, China) [22]. The *Zea mays* L. seedlings were planted to propagate the inoculants comprising sand, spores (about 50 spores/gram), mycelia, and colonized root fragments.

### 2.2. Plant Cultivation and Soil Preparation

Seeds of *Robinia pseudoacacia* L., acquired from a local market (YangLing, China), were surface sterilized with 5% NaClO_3_ solution for 10 min and then rinsed ten times with sterile water [22]. The sterilized *R. pseudoacacia* seeds and sterilized, moistened filter paper were placed in petri dishes to germinate. The germinated seeds were transferred to seedling trays that were pre-sterilized in a 1% KMnO_4_ solution and filled with nursery substrates sterilized at 121 °C for 2 h, after which the seedlings were watered daily with sterilized water. After 2 weeks in the greenhouse (a relative humidity of 65–75% and temperatures of 26 °C) of Northwest A&F University, seedlings with consistent growth were selected as experimental materials and were transplanted into plastic pots (10 cm × 12 cm) containing 500 g of substrate. The mixture of fine sand (<2 mm) and vermiculite (1:1, *v*/*v*), used in this study, was autoclaved (0.11 MPa, 121 °C) for 2 h. The sand was rinsed under running water until the runoff was clear.

### 2.3. Experimental Design and Plant Growth

An experiment was carried out in the climate chamber of Northwest A&F University (Yangling, China) with 65–75% humidity and day/night temperature (26 °C/24 °C, 16 h/8 h). The experiment included three factors, Zn amount (0 or 1000 mg Zn kg^−1^ soil), inoculation status (inoculated with *R. irregularis*, AM; non-inoculated with *R. irregularis*, NM), ABA conditions (with or without exogenous ABA addition). Three biological replicates were set up for each treatment. There were 24 pots in all. Each pot comprised one black locust seedling and 500 g of sterile growth substrate. Each AM treatment was inoculated with 10 g of inoculants when the plants were transplanted into the pots, and each non-mycorrhizal treatment was inoculated 10 g of the sterile inoculants with AM inoculant filtrate (<20 µm). The plants were watered every day and fortified with 50 mL of Hoagland solution comprising half-strength phosphorus every 2 weeks throughout the experimental period. After the seedlings grew for 6 weeks in the pots, two different concentrations of Zn solution (0 and 22.1 g L^−1^ ZnSO_4_·7H_2_O) were supplemented into the matching pots for 5 days (20 mL per day; 100 mL was added in total). According to the methods of Shi et al. [13], 10 µM ABA solutions were applied to the culture substance of the ABA-treated plants after seedlings grew for 9 weeks in pots. The same amount of ddH_2_O was applied to plants not treated with ABA. The ABA treatment lasted for one week [13], after which the plants were harvested (Figure S1).

### 2.4. Plant Sampling and Biomass Measurements

Ten weeks after the seedlings were inoculated with AM fungi, the leaves, stems and roots of each plant were sampled, separately. The fresh shoots (consisted of leaves and stems) and roots were calculated. A portion of the samples were dried to obtain dry weight (DW) and Zn measurements. A portion of the roots, which were fixed in FAA solution (consisted of 37% formaldehyde, glacial acetic acid and 95% ethanol, 9:0.5:0.5, *v*/*v*/*v*), were used to determine mycorrhizal fungal colonization [23]. The rest of samples were ground to powders in liquid nitrogen and cryopreserved at −80 °C until use.

### 2.5. AM Colonization

The roots fixed in FAA solution were cut into pieces (1 cm length), clarified in 5% KOH (*w*/*v*) at 90 °C for 2 h, then acidified with 1% HCl, stained with trypan blue (0.05%) in lactophenol [24]. The method of gridline intersect was used to estimate AM fungal colonization, according to McGonigle et al. [25].

### 2.6. Analysis of Abscisic Acid Contents

According to the modified methods of Shi et al. [13], ABA was extracted with ice-cold 80% (1 mL, *v*/*v*) methanol that included 200 mg L^−1^ butylated hydroxytoluene and shaken overnight at 4 °C. The supernatants were collected via centrifugation at 8000× *g* and 4 °C for 10 min. The residues were resuspended for 2 h, and the supernatants were combined. The extracts were then dried under N_2_ and redissolved in 0.5 mL of a mobile phase solution until measurements were performed. The ABA contents in the extracts were determined with a 1260 high-performance liquid chromatography system (Agilent, Palo Alto, CA, USA). ABA ((±) −ABA, A1049, Sigma, St. Louis, MO, USA) was used as a standard to calculate the ABA contents.

### 2.7. Analysis of Total Zn Contents and Water-Soluble Zn Complex Contents

Oven-dried shoot and root samples were ground with a mortar and screened with a 0.5-mm sieve. The resulting fine powder samples (0.1 g) were digested in 8 mL HNO_3_ + 2 mL HClO_4_ at temperatures slowly increasing to 220 °C. The Zn content in the digested solution were analyzed by a flame atomic absorption spectrophotometer (PinAAcle 500, Waltham, MA, USA). According to the methods of Zeng et al. [26], the water-soluble Zn complex was extracted by deionized water and was estimated using flame atomic absorption spectrophotometer (PinAAcle 500, Waltham, MA, USA).

### 2.8. Analysis of Proline Contents

The proline contents were elevated according to Bates et al. [27]. The fine powder samples of the roots (0.2 g) were extracted in 3% sulfosalicylic acid (5 mL, *w*/*v*) in a boiling water bath for 15 min and then were centrifuged at 12,000× *g* for 10 min. The supernatants were then collected to evaluated at the wavelengths of 520 nm.

### 2.9. Analysis of P5CS, ProDH and OAT Activities

Fine powder samples of roots were extracted using 50 mM Tris-HCl buffer (pH 7.4) comprising 7 mM MgCl_2_, 3 mM EDTA, and 5% (*w*/*v*) insoluble PVP to analyze the activities of Δ^1^-Pyrroline-5-carboxylate synthetase (P5CS) and proline dehydrogenase (ProDH) [28]. After centrifugation at 12,000× *g* and 4 °C for 20 min, the supernatants were collected to determine the P5CS and ProDH activities. The supernatants (1 mL) were added to a reaction mixture (1 mL, pH 7.0) consisting of 50 mM Tris-HCl, 20 mM MgCl_2_, 100 mM hydroxamate-HCl, 10 mM ATP, and 50 mM L-glutamate, and then heated in a 37 °C water bath for 15 min. The stop buffer (comprising 5 M HCl, 2.5% FeCl_3_, and 12% trichloroacetic acid (TCA)) was added to the mixture to stop the reaction; the solution was then centrifuged for 15 min at 12,000× *g*. The supernatant was subsequently used for measuring the activity of P5CS, and reactions without ATP constituted the blank controls. The activity of ProDH was assessed by monitoring the decrease of NAD^+^ (or NADP^+^) according to the methods of Sánchez et al. [28].

Following the methods of Sánchez et al. [28], root samples were extracted and powdered to measure the activity of ornithine-δ-aminotransferase (OAT) using a solution consisting of 50 mM potassium phosphate buffer (pH 7.9), 1 mM EDTA and 15% glycerol. The activity of OAT was evaluated by monitoring the reduction of NADH.

### 2.10. Analysis of Lipid Peroxidation and Reactive Oxygen Species (ROS)

The contents of malondialdehyde (MDA) were used to evaluate the degree of lipid peroxidation. According to the methods of Kumar and Knowles [29], 0.1 g of powdered samples of roots were extracted using 5% TCA (1 mL). After centrifugation for 10 min at 12,000× *g*, the supernatant and thiobarbituric acid (TBA) were mixed together and then heated in a boiling water bath for 15 min. The mixture was cooled rapidly in running tap water and then centrifuged at 5000× *g* for 10 min. The supernatant was collected to evaluate the wavelengths of 450 nm, 532 nm, and 600 nm.

The generation rate of O_2_^·−^ was measured following the methods of Ke et al. [30]. Powdered root samples were extracted with 0.05 M ice-cold potassium phosphate buffer (pH 7.8), and then centrifuged for 20 min at 12,000× *g* and 4 °C. The supernatants were collected to assay the production rate of O_2_^·−^.

The H_2_O_2_ contents of the roots were determined by monitoring the reaction with potassium iodide (KI) using spectrophotometric method, as described by Chakrabarty and Datta [31]. Fine powder samples of the roots were homogenized using 0.1% (*w*/*v*) TCA and then centrifuged for 20 min at 15,000× *g*. The supernatant was collected for measurements of the H_2_O_2_ contents, and 100 μM H_2_O_2_ was used as a standard.

### 2.11. Analysis of Antioxidant Enzyme Activities

Powdered root samples were homogenized with ice-cold extraction buffer (pH 7.8) comprising 0.05 M phosphate, 1% PVP (*w*/*v*), and 1 mM EDTA-Na_2_. After being centrifuged for 20 min at 12,000× *g* and 4 °C, and the supernatant was used as a crude enzyme mixture. The total soluble protein content was measured by the colorimetric method with Coomassie brilliant blue G250, and the wavelength for determination was 595 nm [32].

Superoxide dismutase (SOD) activity was estimated by monitoring the inhibition of the photochemical reduction of nitro blue tetrazolium (NBT) [32]. According to the methods of Gao [32], the catalase (CAT) activity was assayed by monitoring the rate of decomposition of H_2_O_2_ during 3 min, and the wavelength for determination was 240 nm. The activity of peroxidase (POD) was measured by assaying the oxidation of guaiacol to tetraguaiacol [32]. Crude enzyme extract (0.3 mL) and 0.05 M phosphate buffer (pH 6.0, consisting of 0.01% guaiacol, 6 mM H_2_O_2_) were mixed together, and the increase of optical density at 470 nm was recorded. The activity of ascorbate peroxidase (APX) was evaluated by monitoring the rate of ascorbate oxidation, which was determined by the wavelength of 290 nm [33]. The assay was performed in a reaction mixture (3 mL) of the crude enzyme extract and 0.05 M phosphate buffer (pH 7.0, 20 °C, comprising 88.3 μM EDTA-Na_2_, 250 μM ascorbate, and 1 mM H_2_O_2_).

### 2.12. Statistical Analysis

The SPSS 21.0 software package (SPSS, Inc., Chicago, IL, USA) was used to carry out statistical tests. Three-way ANOVA was used to analyze the data followed by application of Tukey’s test when ANOVA indicated a remarkable difference. Pearson’s tests (*p* < 0.05) were used to perform correlation analysis.

## 3. Results

### 3.1. Biomass and Colonization

**Figure 1:**
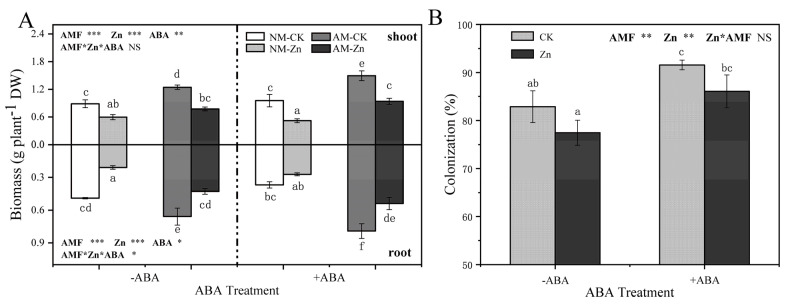
(**A**) Dry weights of shoots and roots of AM plants grown under either control or Zn stress and treated with either 0 (-ABA) or 10 µM ABA (+ABA) application. NM-CK: non-AM inoculation and 0 mg kg^−1^ Zn treatment; NM-Zn: non-AM inoculation and 1000 mg kg^−1^ Zn treatment; AM-CK: AM inoculation and 0 mg kg^−1^ Zn treatment; AM-Zn: AM inoculation and 1000 mg kg^−1^ Zn treatment. (**B**) AM colonization of black locust under Zn stress and ABA addition. CK: 0 mg kg^−1^ Zn treatment, Zn: 1000 mg kg^−1^ Zn treatment. The data show the means ± standard deviations (*n* = 3). The different letters above the bars indicate significant differences among the treatments according to Tukey’s test: (*p* < 0.05). *, *p* < 0.05; **, *p* < 0.01; ***, *p* < 0.001; NS, not significant.

Compared with no Zn stress, Zn stress notably decreased the dry weight of the shoots and roots (Figure 1A). AM inoculation led to 29–80% and 34–115% increase in the shoot and root biomass of *R. pseudoacacia* L., respectively. Under Zn stress, exogenous ABA application slightly enhanced the biomass of mycorrhizal plants.

No mycorrhizal roots were observed in NM plants. Regardless of ABA addition, AM colonization was slightly decreased by Zn stress (Figure 1B). Regardless of Zn stress, exogenous ABA application positively enhanced AM colonization. Moreover, AM plants with ABA application presented the highest colonization-up to 92%.

### 3.2. Total Zn Contents and Water-Soluble Zn Complex Contents

**Figure 2:**
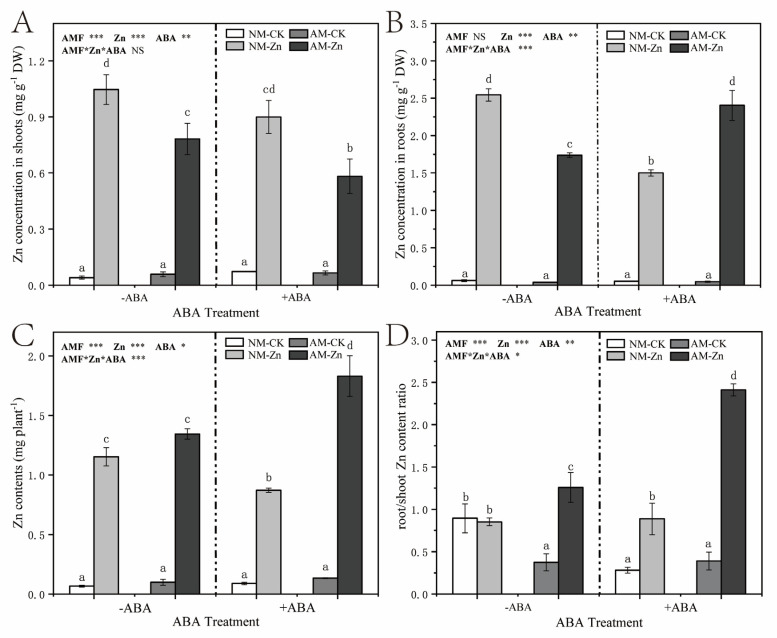
(**A**,**B**) Zn concentration in the shoots and roots of black locust plants; (**C**) Total Zn contents in plants; (**D**) root/shoot Zn content ratio of black locust plants. The data show the means ± standard deviations (*n* = 3). The different letters above the bars indicate significant differences among the means of the Zn concentration in the shoots, the Zn concentration in the roots, the total Zn content or the ratio of root/shoot and Zn content ratio according to Tukey’s test (*p* < 0.05). *, *p* < 0.05; **, *p* < 0.01; ***, *p* < 0.001; NS, not significant. The abbreviations used are the same as those used above.

Compared with no Zn stress, Zn stress dramatically raised the Zn concentration and total Zn content of black locust (Figure 2). In the shoots, under Zn stress, AM inoculation and ABA application strongly decreased the Zn concentration. In the roots exposed to Zn stress, inoculation with AM fungi markedly reduced Zn concentrations under 0 µM ABA treatment, whereas the trends were opposite under the 10 µM ABA treatment. With Zn stress, ABA application notably enhanced the Zn concentration of AM roots, and yet it reduced the Zn concentration of NM roots. In addition, under Zn stress, AM plants showed a higher Zn content than the NM plants (an increase of 17–110%). ABA application increased the total Zn content of AM seedlings, and the contrasting trends were observed in NM plants.

The root/shoot Zn content ratio increased in plants in response to Zn stress in most treatments, except in NM plants without ABA application (Figure 2D). Exposed to Zn stress, AM inoculation increased root/shoot to Zn content ratio by 47% and 172% in the absence and presence of exogenous ABA, respectively. Under Zn stress, in NM plants, exogenous ABA application had no remarkable effect on the root/shoot to Zn content ratio compared with that of plants under the 0 µM ABA treatment; however, in AM plants, exogenous ABA largely increased the root/shoot to Zn content ratio by 92%.

Table S1 shows that the water-soluble Zn complex contents were largely increased by Zn stress in all of issues studied. Under Zn stress, AM inoculation dramatically decreased the water-soluble Zn complex contents both in the leaves, stems and roots, by 54–56%, 39–63%, and 50–67%, respectively. Under Zn stress, in AM roots, exogenous ABA application observably decreased the water-soluble Zn complex; whereas in NM roots, no similar trend was observed.

### 3.3. ABA Contents

In the leaves, Zn stress decreased the ABA concentration in the AM treatment but increased the ABA concentration in the NM treatment (Table 1). Without Zn stress, AM inoculation enhanced the ABA concentration to varying degrees; whereas with Zn stress, AM inoculation had a negative effect on the ABA concentration. In the presence of Zn stress, exogenous ABA application markedly increased the ABA concentration in NM leaves but did not affect ABA concentration in AM leaves.

In the roots, ABA concentration was unaffected by Zn stress except in NM roots without ABA application. Without Zn stress, AM inoculation did not affect ABA concentration in the 0 and 10 µM ABA treatment. With Zn stress, AM inoculation largely increased ABA concentration. Under Zn stress, exogenous ABA application significantly increased ABA concentration in NM roots, but did not affect ABA concentration in AM roots.

Zn stress decreased the root/leaf to ABA content ratio in NM plants, but increased in AM plants. With Zn stress, AM inoculation increased the root/leaf to ABA content ratio, compared to NM plants, by 63% and 152% with and without ABA application, respectively. Under Zn stress, exogenous ABA application increased the root/leaf to ABA content ratio in AM plants but had no significant effect on the root/leaf to ABA content ratio in NM plants.

### 3.4. Proline Contents and Proline Metabolism-Related Enzyme Activity

**Figure 3:**
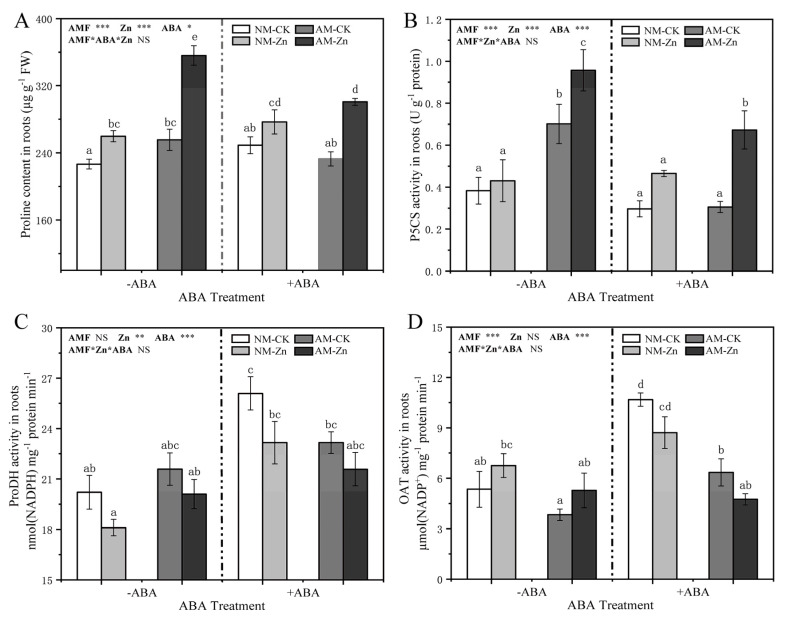
(**A**–**D**) Proline content, P5CS activity, ProDH activity, and OAT activity in roots. The data show the means ± standard deviations (*n* = 3). The different letters above the bars indicate significant differences among the means of the proline content, P5CS activity, ProDH activity, and OAT activity according to Tukey’s test (*p* < 0.05). *, *p* < 0.05; **, *p* < 0.01; ***, *p* < 0.001; NS, not significant. The abbreviations used are the same as those used above.

The proline contents were sharply increased by Zn stress in all treatments (Figure 3A). AM inoculation significantly increased the proline contents by 8.0–37% compared with NM roots, except in the 0 mg kg^−1^ Zn treatment with ABA application. Regardless of Zn stress, ABA application increased the proline contents of NM roots, but the opposite trends were observed in AM roots. In addition, proline content was dramatically related to P5CS activity (*r* = 0.748, *p* < 0.001) (Figure S2A). However, there were no significant correlations between proline contents and ProDH activity or OAT activity (*r* = −0.135, *p* = 0.53; *r* = −0.141, *p* = 0.511) (Figure S2B,C).

In roots, Zn stress increased P5CS activity to varying degrees (Figure 3A). In the roots suffered to Zn stress, compared with non-mycorrhizal treatments, AM inoculation dramatically enhanced the activity of P5CS with and without ABA application (increases of 46% and 122%, respectively). Under Zn exposure, exogenous ABA addition had a negative effect on P5CS activity in AM roots, but no similar phenomenon was detected in NM roots. Regardless of the presence of Zn stress, ProDH activity in NM roots significantly increased (by 27–30%) in response to ABA application compared with 0 µM ABA treatment. However, exogenous ABA application had a slightly positive effect on ProDH activity in AM roots. Figure 3D shows that the OAT activity was slightly increased by Zn stress without exogenous ABA addition, while the opposite trends were observed in the presence of ABA. Compared to NM roots, inoculation with AM fungi reduced OAT activity to varying degrees. In addition, under Zn exposure, exogenous ABA application increased the OAT activity in NM roots, but slightly reduced the OAT activity in AM roots.

### 3.5. Antioxidant System Parameters

In roots, the O_2_^·−^ generation rate and the MDA contents were greatly increased due to Zn stress (Table 2). In addition, Zn stress increased the H_2_O_2_ contents compared with non-Zn treatment in AM roots. Regardless of ABA application, inoculation with *R. irregularis* notably reduced MDA contents ranging from 35–67% compared with NM roots. Inoculation with AM fungi decreased the O_2_^·−^ generation rate by 61–67% and 25–51% without and with ABA treatment, respectively. In the roots exposed to Zn stress, AM inoculation slightly reduced the contents of H_2_O_2_. Exogenous ABA application decreased the O_2_^·−^ generation rate and the contents of H_2_O_2_ to varying degrees in roots exposed to Zn stress. Under Zn exposure, ABA application clearly decreased the MDA contents in mycorrhizal roots, but no similar trends were observed in non-mycorrhizal roots.

Regardless of ABA application, Zn stress enhanced the activities of SOD and POD. The CAT and APX activities were increased by Zn stress in the roots without ABA application, but the trends were opposite in the roots with ABA application. Compared with NM roots, inoculation with AM fungi increased the activities of SOD and CAT by 18–43% and 20–41%, respectively (Table 2). The activities of APX and POD were also slightly increased after AM inoculation. In the roots exposed to Zn stress, ABA application increased SOD and POD activities, but reduced CAT and APX activities. AM roots with ABA and Zn applications had the highest SOD and POD activities among those of all the treatments.

### 3.6. The Correlation Analysis

**Figure 4:**
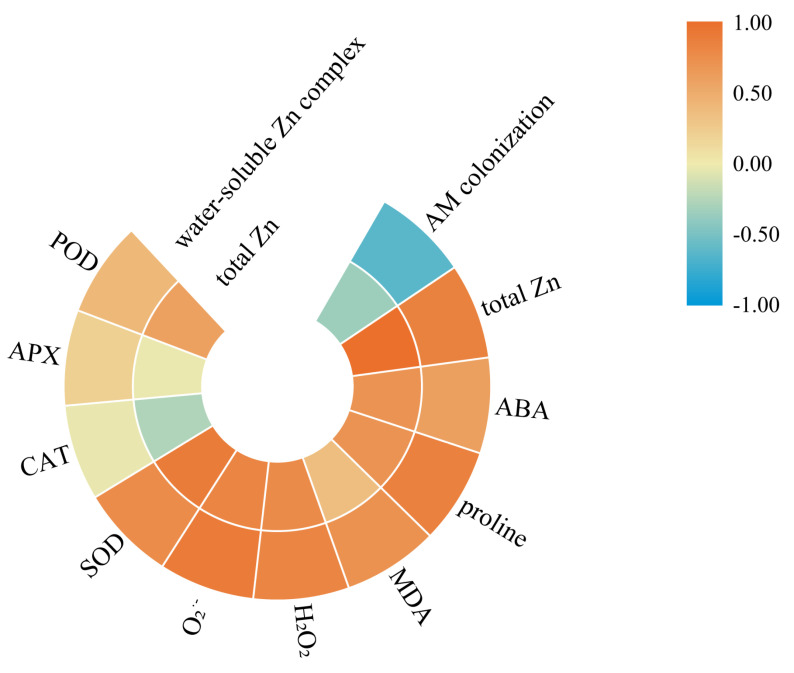
The relationships between water-soluble Zn complex contents, total Zn contents and AM colonization, total Zn contents, ABA contents, proline contents, MDA contents, H_2_O_2_ contents, O_2_^·−^ generation rate, SOD activity, CAT activity, APX activity, and POD activity. Pearson’s correlation analysis was performed at the levels of 5%. *n* = 12.

To determine the response patterns of black locusts inoculated with AM to Zn stress, a correlation analysis was performed using data related to Zn contents, ABA contents, osmo-protectants, ROS, and antioxidants (Figure 4). In AM plants, there was a sharp negative correlation between AM colonization and water-soluble Zn complex contents (*r* = −0.628, *p* = 0.029), but no significant correlation was observed between AM colonization and the total Zn contents (*r* = −0.345, *p* = 0.272). In addition, Figure 4 shows that ABA contents were markedly positively compared to water-soluble Zn complex content as well as total Zn content in AM roots. Interestingly, the correlations between the water-soluble Zn complex content and proline content, MDA content, ROS levels, and SOD activity were stronger than those between the total Zn content and the indices of these antioxidants.

## 4. Discussion

### 4.1. ABA Enhanced Excess Zn Tolerance by Improving Arbuscular Mycorrhizal Symbiosis

Biomass production was the most distinct trait that reflected the plant performance in response to abiotic stress and the effects of AM symbiosis on hosts [34]. Mycorrhizal black locusts grew better than non-mycorrhizal black locusts under Zn stress, which indicated that the inoculation of AM fungi could ameliorate Zn stress tolerance in black locust (Figure 1A). Favorable effects of AM fungi inoculation on the biomass of plants growing under heavy metal stress were also found in other plants of the legume variety, such as soybean and alfalfa [19,23]. AM fungi prodcued high plant biomass via a reduction in the toxicity of heavy metals [35,36]. However, heavy metals were toxic to plant roots and could reduce or even inhibit the symbiosis efficiency of AM fungi, which negatively effected the symbiosis between plant roots and AM fungi [23]. In the present study, high levels of Zn reduced mycorrhizal colonization, which was consistent with the results of previous research on tomato [37]. We also found that exogenous ABA application promoted the growth of plants with mycorrhizal fungi and AM fungal colonization under a 1000 mg kg^−1^ Zn condition, which indicated that exogenous ABA might modulate the colonization of *R. irregularis* in black locust roots and result in a stronger AM symbiosis. Martín-Rodríguez et al. [21] observed that the inhibition of ABA biosynthesis negatively affected parameters related to mycorrhization and that exogenous ABA application rescued arbuscular fungi abundance in mycorrhizal roots, which suggested that ABA could regulate the colonization of plants by AM fungi. Previous studies have shown that ABA played an important role in the development of the complete arbuscule and its functionality [20]. Therefore, these results suggested that exogenously applied ABA alleviated the toxic effects of excess Zn to AM plants and enhanced the Zn tolerance of AM plants by improving AM symbiosis.

### 4.2. ABA Played Different Roles in AM and Non-AM Plants under Zn Stress

ABA is a vital signal transduction phytohormone and plays a pivotal role in response to various abiotic stresses, including Zn stress [13]. In response to abiotic stress, the variations in endogenous ABA content were different in non-mycorrhizal and mycorrhizal plants, which resulted in different ways through which NM plants and AM plants acclimate to adverse environments [38]. In NM plants, the endogenous ABA contents increased in the leaves in response to Zn but decreased in the roots (Table 1). Previous studies demonstrated that endogenous ABA accumulation in the leaves contributed to promoting tolerance to heavy metal stress in NM plants [13,39]. Zhu et al. [7] suggested that the combined use of ABA and salicylic acid could promote Cd accumulation in senescent leaves to prevent young leaves from injury caused by Cd stress. These results suggested that NM plants may have coped with Zn toxicity by increasing ABA accumulation in the leaves, which was unfavorable to the growth of aerial plant parts. This was consistent with the reduction in the biomass of shoots in NM plants (Figure 1A). Interestingly, in AM plants, Zn stress increased the root/leaf to ABA content ratios (Table 1). Higher ABA contents were reported as occurring in AM roots under drought stress, which accelerated the growth of roots inoculated with mycorrhizal fungi [10]. In addition, Ren et al. [40] observed that inoculation with AM fungi could reduce leaf ABA levels in *Zea mays* L. exposed to drought, which contributed to maintaining aerial growth. These results demonstrated that mycorrhizal plants had a greater survival potential in response to Zn stress than non-mycorrhizal plants due to the increased proportion of ABA in the roots.

Decreasing the transport of heavy metals to the shoots is one of the most vital mechanisms by which plants alleviate heavy metal phytotoxicity [4,34]. We found that under Zn stress, the root/shoot Zn content ratios were significantly increased by AM inoculation regardless of ABA application (Figure 2D), indicating that AM fungi may be involved in the restriction of Zn transport from the roots to the aboveground parts. AM fungal structure was reported to accumulate high heavy metal content to reduce heavy metal translocation to the host plant [36,41]. Watts-Williams [42] found that arbuscular mycorrhizal fungi can enhance the retention of HMs in the roots to restrict the translocation to the aerial parts, which might be due to excess Zn accumulating in fungal tissues. In addition, ABA participates in the regulation of acquisition and transport of heavy metals in plants and plays an essential function in ameliorating toxicity effects caused by heavy metals [39]. Interestingly, we found that exogenous ABA addition increased the root/shoot Zn content ratio in AM plants, while it did not affect the root/shoot Zn content ratio in NM plants (Figure 2D). Based on the above findings, we considered that the application of exogenous ABA could stimulate more Zn retention in the roots of AM plants by enhancing AM symbiosis. In our study, the addition of exogenous ABA significantly increased AM colonization under Zn stress, which could explain our analysis. Exogenous ABA application was also shown to alleviate Cd toxicity by preventing Cd transport from the roots to the shoots of purple-flowering stalk seedlings [43]. Therefore, our results showed that AM fungi and ABA could exert protective effects on host plants under Zn stress, which may be due to the increased retainment of Zn in mycorrhizal roots, thus protecting aerial parts from damage caused by excess Zn.

### 4.3. ABA Application Stimulated the Antioxidant Response of Mycorrhizal Plants

Excess Zn was reported to induce oxidative stress in higher plants, resulting in an imbalance between ROS and antioxidants [44]. In the current study, both ABA application and AM inoculation decreased the contents of MDA and H_2_O_2_ and the O_2_^·−^ generation rate to prevent black locust from experiencing oxidative damage, which could explain why AM roots accumulated higher levels of Zn but experienced less damage than NM roots [45]. Previous research has also suggested that the increased activities of antioxidative enzymes, acting as vital scavengers to eliminate ROS, contributed to protecting plants from injury caused by excess Zn [13,36]. In response to Zn stress, AM inoculation increased SOD activity, which demonstrated that AM inoculation is in favor of scavenging O_2_^·−^ radicals [23]. It is necessary to rapidly and efficiently reduce the excessive H_2_O_2_ after SOD catalyzes the dismutation of O_2_^·−^ to H_2_O_2_ and O_2_. H_2_O_2_ is detoxified by CAT, POD and APX [46]. In our study, AM inoculation improved the CAT, POD and APX activities with Zn stress, and ABA application increased the POD activity. These results revealed that ABA addition and AM inoculation increased H_2_O_2_ detoxification. Therefore, AM fungi and ABA could exert protective effects on the Zn tolerance of host plants through decreasing oxidative damage and increasing antioxidant enzyme activity.

In addition, proline was a common organic osmo-protectant that provided a protection against heavy metal-induced oxidative stress [47,48]. In this study, under Zn stress, the content of proline in mycorrhizal and non-mycorrhizal plants had different responses to exogenous ABA application. Under Zn stress conditions, exogenous ABA addition increased the proline contents in NM roots. Similarly, increased proline contents were previously reported in non-mycorrhizal Arabidopsis thaliana [48]. However, in AM roots exposed to Zn stress, reduced proline contents were observed in response to exogenous ABA application. The reason for this finding may be that the addition of exogenous ABA notably reduced the content of the water-soluble Zn complex in mycorrhizal roots, which might have reduced the toxic effects of excess Zn on black locust. These results were consistent with the results of a significant positive correlation between water-soluble Zn complex content and proline content in AM roots (Figure 4). In general, after heavy metals entered the roots, they were present in various chemical species, and water-soluble heavy metal complexes were one of the main forms with deleterious effects on plant cells [49]. Therefore, there was no need more proline to cope with Zn stress. Correlation analysis revealed that the activity of P5CS was positively correlated with ABA content, indicating that P5CS played a vital role in proline accumulation. It is well known that Δ^1^-pyrroline-5-carboxylate synthetase (P5CS) regulated proline synthesis and had a rate-limiting function in proline accumulation [50]. Overall, ABA application employed different methods to increase Zn tolerance in NM or AM plants: ABA application enhanced Zn stress tolerance by increasing proline contents in NM plants or by decreasing the water-soluble Zn complex contents in AM plants.

## 5. Conclusions

AM inoculation ameliorated the effects of excess Zn toxicity, which may be achieved through stimulating the accumulation of abscisic acid in the roots. Exogenous ABA application shifted the strategy of Zn stress tolerance in AM plants; that is, exogenous ABA promoted excess Zn retention in the roots by enhancing AM symbiosis, which protected the leaves of AM plants and improved the growth of AM plants. In addition, the amelioration of oxidative damage by AM inoculation and ABA application was related to a reduction in the water-soluble Zn complex, higher organic osmo-protectants, and an activated antioxidant defense system. In summary, this study shows that improved AM symbiosis, altered the distribution of Zn and stimulated the antioxidant defense system induced by ABA addition may be a strategy to improve the growth of plants and their tolerance to Zn stress. Therefore, host plants inoculated with AM fungi and simultaneously applied with exogenous ABA had better potential to alleviate heavy metal stress.

## Figures and Tables

**Table 1:** The ABA concentrations in the leaves and roots and the root/leaf to ABA content ratio.

ABA Treatment	Inoculation Treatment	Zinc Treatment	Leaves (µg g^−1^ FW)	Roots (µg g^−1^ FW)	Root/Leaf of ABA Content Ration
−ABA	NM	Zn0	0.66 ± 0.13 a	1.15 ± 0.11 bc	4.69 ± 0.25 d
		Zn1000	0.94 ± 0.11 bc	0.74 ± 0.06 a	1.83 ± 0.21 a
	AM	Zn0	1.07 ± 0.04 c	1.10 ± 0.02 bc	2.45 ± 0.22 ab
		Zn1000	0.73 ± 0.12 ab	1.30 ± 0.02 bc	2.98 ± 0.44 b
+ABA	NM	Zn0	0.61 ± 0.04 a	1.22 ± 0.13 bc	3.86 ± 0.30 c
		Zn1000	1.53 ± 0.08 d	1.03 ± 0.22 b	1.90 ± 0.30 a
	AM	Zn0	0.78 ± 0.06 ab	1.21 ± 0.15 bc	2.91 ± 0.31 b
		Zn1000	0.69 ± 0.03 a	1.34 ± 0.12 c	4.78 ± 0.11 d
Significance					
AMF			**	**	NS
Zn			***	NS	***
ABA			NS	*	**
AMF*Zn			***	***	***
AMF*ABA			***	NS	***
ABA*Zn			***	NS	***
AMF*Zn*ABA			*	NS	NS

The data show the means ± standard deviations (*n* = 3). The different letters above the bars indicate significant differences among the means of the ABA contents in the leaves and roots and the root/shoot to ABA content ratio according to Tukey’s test (*p* < 0.05). Significant effects of three-way ANOVA: *, *p* < 0.05; **, *p* < 0.01; ***, *p* < 0.001; NS, not significant. -ABA: 0 µM ABA application; + ABA: 10 µM ABA application; AM: AM fungal inoculation; NM: non-AM fungal inoculation; Zn0: 0 mg kg^−1^ Zn treatment; Zn1000: 1000 mg kg^−1^ Zn treatment; AMF: AM fungal colonization; Zn: Zn stress; ABA: ABA application.

**Table 2:** Contents of MDA and H_2_O_2_, O_2_^·−^ generation rate, and the activity of antioxidant enzymes.

Treatment			MDA (µmol g^−1^ FW)	H_2_O_2_ (µmol g^−1^ FW)	O_2_^·−^ Generation Rate (nmol min^−1^ g^−1^ Protein)	SOD Activity (U mg^−1^ Protein)	CAT Activity (U mg^−1^ Protein)	POD Activity (U mg^−1^ Protein)	APX Activity (U mg^−1^ Protein)
−ABA	NM	Zn0	30.67 ± 1.42 b	7.73 ± 0.73 bc	384.15 ± 22.46 d	1.52 ± 0.30 a	1.70 ± 0.05 a	57.24 ± 5.08 a	1.60 ± 0.11 a
		Zn1000	54.07 ± 4.99 c	8.44 ± 0.42 c	520.52 ± 17.32 e	2.16 ± 0.07 ab	2.87 ± 0.08 cd	71.64 ± 0.98 bc	2.01 ± 0.04 cd
	AM	Zn0	17.94 ± 1.79 a	6.62 ± 0.049 ab	129.78 ± 20.38 a	2.11 ± 0.15 ab	2.39 ± 0.13 abc	65.33 ± 6.74 ab	1.73 ± 0.06 ab
		Zn1000	27.83 ± 4.47 b	7.89 ± 0.57 c	202.54 ± 29.38 bc	3.00 ± 0.36 cd	3.47 ± 0.56 de	82.46 ± 2.79 d	2.23 ± 0.02 d
+ABA	NM	Zn0	23.83 ± 1.03 ab	8.19 ± 0.25 c	238.14 ± 16.18 c	2.05 ± 0.12 ab	3.05 ± 0.42 cd	66.61 ± 0.84 ab	1.90 ± 0.13 bc
		Zn1000	56.00 ± 3.27 c	7.93 ± 0.14 c	246.28 ± 7.88 c	2.32 ± 0.35 bc	1.86 ± 0.07 ab	80.25 ± 0.51 cd	1.63 ± 0.04 a
	AM	Zn0	15.60 ± 1.55 a	5.66 ± 0.55 a	116.77 ± 15.54 a	2.42 ± 0.15 bc	4.21 ± 0.38 e	86.84 ± 3.61 de	2.22 ± 0.18 d
		Zn1000	17.05 ± 2.99 a	7.49 ± 0.21 bc	182.66 ± 8.52 b	3.30 ± 0.27 d	2.57 ± 0.09 bc	101.77 ± 2.34 e	1.82 ± 0.03 abc
Significance									
AMF			***	***	***	***	***	***	***
Zn			***	***	***	***	NS	***	NS
ABA			**	NS	***	*	*	***	NS
AMF*Zn			***	**	NS	NS	NS	NS	NS
AMF*ABA			NS	NS	***	NS	NS	*	NS
ABA*Zn			NS	NS	***	NS	***	NS	***
AMF*Zn*ABA			**	*	**	NS	NS	NS	NS

The data show the means ± standard deviations (*n* = 3). The different letters within each column indicate significant differences among the means according to Tukey’s test (*p* < 0.05). Significant effects of three-way ANOVA: *, *p* < 0.05; **, *p* < 0.01; ***, *p* < 0.001; NS, not significant. The abbreviations used are the same as those used above.

## Data Availability

Not applicable.

## Supplementary Materials

The following are available online at https://www.mdpi.com/article/10.3390/jof7080671/s1, Figure S1: A schematic diagram illustrating the experimental design of this study; Figure S2: The relationships between proline content and P5CS activity, ProDH activity, OAT activity; Table S1: Water-soluble Zn complex contents in the leaves, stems and roots.
