# Supplementary material for: The Synergy of Arbuscular Mycorrhizal Fungi and Exogenous Abscisic Acid Benefits Robinia pseudoacacia L. Growth through Altering the Distribution of Zn and Endogenous Abscisic Acid
Source: J Fungi (Basel). 2021 Aug 19;7(8):671. doi: 10.3390/jof7080671 (PMC8400989; doi:10.3390/jof7080671)
Supplement: Supplementary file 1 [file jof-07-00671-s001.zip › jof-1313191-supplementary/supplementary/jof-1313191 table s1 figure s1 s2.pdf]

Supporting information to:

The Synergy of Arbuscular Mycorrhizal Fungi and Exogenous Abscisic Acid Benefits *Robinia pseudoacacia* L. Growth through Altering the Distribution of Zn and Endogenous Abscisic Acid

Xiao Lou, Xiangyu Zhang, Yu Zhang, Ming Tang *
*Correspondence: **Ming Tang**
Email address: tangm@nwsuaf.edu.cn

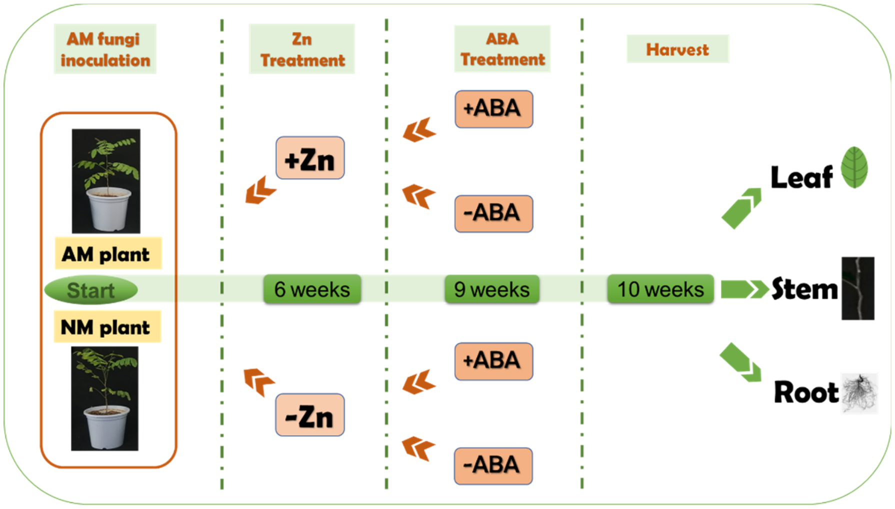

Supplementary Fig. S1 A schematic diagram illustrating the experimental design of this study. AM plant: plants inoculated with AM fungi; NM: plants inoculated without AM fungi; +Zn: 0 mg Zn kg^{-1} soil; -Zn: 1000 mg Zn kg^{-1} soil; +ABA: 10 μM ABA application; -ABA: 0 μM ABA application.

Table S1 Water-soluble Zn complex contents in the leaves, stems and roots.

ABA Treatment	Inoculation Treatment	Zinc Treatment	Leaves ($\mu g\ g^{-1}$ DW)	Stems ($\mu g\ g^{-1}$ DW)	Roots ($\mu g\ g^{-1}$ DW)
–ABA	NM	Zn0	15.47±1.11a	7.18±0.75a	2.99±0.28a
		Zn1000	280.96±0.25c	75.28±3.54d	197.88±19.03d
	AM	Zn0	11.07±0.21a	9.74±0.75a	5.90±0.99a
		Zn1000	128.50±4.44b	28.56±2.37b	97.93±7.66c
+ABA	NM	Zn0	15.17±2.06a	6.74±1.85a	4.31±2.27a
		Zn1000	305.46±12.34d	55.68±7.78c	190.31±8.87d
	AM	Zn0	11.82±1.09a	11.58±0.72a	6.25±0.86a
		Zn1000	134.93±12.98b	33.94±5.50b	62.84±1.19b
	Significance				
	AMF		***	***	***
	Zn		***	***	***
	ABA		**	NS	**
	AMF*Zn		***	***	***
	AMF*ABA		NS	***	*
	ABA*Zn		*	*	**
	AMF*Zn*ABA		NS	**	NS

The data show the means ± standard deviations (n = 3). The different letters within each column indicate significant differences among the means of the water-soluble water Zn complex contents in the leaves, stems, and roots according to Duncan's test ($P < 0.05$). Significant effects of three-way ANOVA: *, $P < 0.05$; **, $P < 0.01$; ***, $P < 0.001$; NS, not significant. -ABA: 0 μM ABA application; +ABA: 10 μM ABA application; AM: AM fungal inoculation; NM: non-AM fungal inoculation; Zn0: 0 mg kg^{-1} Zn treatment; Zn1000: 1000 mg kg^{-1} Zn treatment. AMF: AM fungal colonization; Zn: Zn stress; ABA: ABA application.

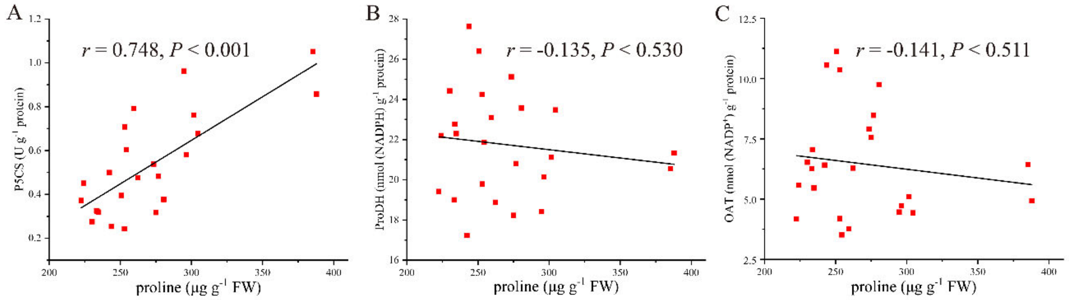

Supplementary Fig. S2 The relationships between proline content and P5CS activity, ProDH activity, OAT activity. Pearson's correlation analysis was made at the levels of 5%. $n = 24$.
