# Supplementary material for: The Synergy of Arbuscular Mycorrhizal Fungi and Exogenous Abscisic Acid Benefits Robinia pseudoacacia L. Growth through Altering the Distribution of Zn and Endogenous Abscisic Acid
Source: J Fungi (Basel). 2021 Aug 19;7(8):671. doi: 10.3390/jof7080671 (PMC8400989; doi:10.3390/jof7080671)
Supplement: Supplementary file 1 [file jof-07-00671-s001.zip › jof-1313191-supplementary/supplementary/jof-1313191-supplementary.pdf]

**Supporting information to:**

# The Synergy of Arbuscular Mycorrhizal Fungi and Exogenous Abscisic Acid Benefits *Robinia pseudoacacia* L. Growth through Altering the Distribution of Zn and Endogenous Abscisic Acid

Xiao Lou, Xiangyu Zhang, Yu Zhang, Ming Tang *

*Correspondence: **Ming Tang**

Email address: tangm@nwsuaf.edu.cn

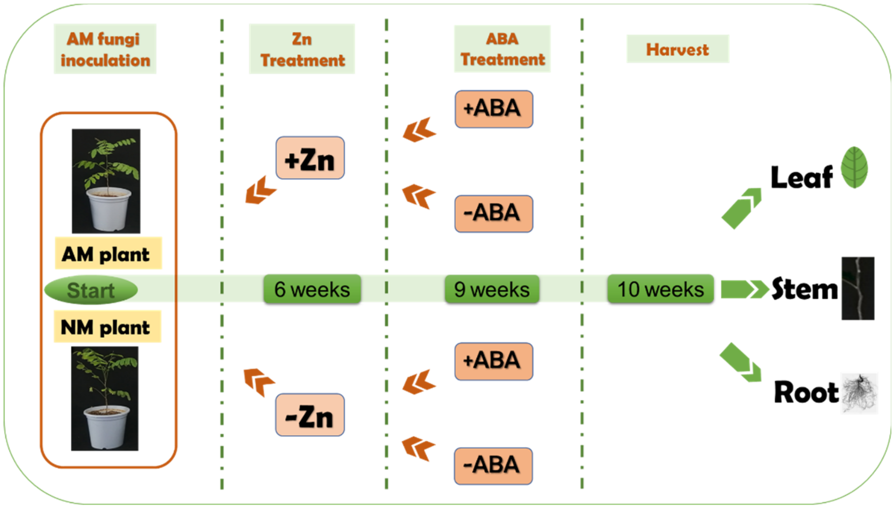


Supplementary Fig. S1 A schematic diagram illustrating the experimental design of this study. AM plant: plants inoculated with AM fungi; NM: plants inoculated without AM fungi; +Zn: 0 mg Zn $kg^{-1}$ soil; -Zn: 1000 mg Zn $kg^{-1}$ soil; +ABA: 10 μM ABA application; -ABA: 0 μM ABA application.

Table S1 Water-soluble Zn complex contents in the leaves, stems and roots.

| ABA Treatment | Inoculation Treatment | Zinc Treatment | Leaves (μg $g^{-1}$ DW) | Stems (μg $g^{-1}$ DW) | Roots (μg $g^{-1}$ DW) |
|---|---|---|---|---|---|
| –ABA | NM | Zn0 | 15.47±1.11a | 7.18±0.75a | 2.99±0.28a |
| | | Zn1000 | 280.96±0.25c | 75.28±3.54d | 197.88±19.03d |
| | AM | Zn0 | 11.07±0.21a | 9.74±0.75a | 5.90±0.99a |
| | | Zn1000 | 128.50±4.44b | 28.56±2.37b | 97.93±7.66c |
| +ABA | NM | Zn0 | 15.17±2.06a | 6.74±1.85a | 4.31±2.27a |
| | | Zn1000 | 305.46±12.34d | 55.68±7.78c | 190.31±8.87d |
| | AM | Zn0 | 11.82±1.09a | 11.58±0.72a | 6.25±0.86a |
| | | Zn1000 | 134.93±12.98b | 33.94±5.50b | 62.84±1.19b |
| | Significance | | | | |
| | AMF | | *** | *** | *** |
| | Zn | | *** | *** | *** |
| | ABA | | ** | NS | ** |
| | AMF*Zn | | *** | *** | *** |
| | AMF*ABA | | NS | *** | * |
| | ABA*Zn | | * | * | ** |
| | AMF*Zn*ABA | | NS | ** | NS |

The data show the means ± standard deviations ($n$ = 3). The different letters within each column indicate significant differences among the means of the water-soluble water Zn complex contents in the leaves, stems, and roots according to Duncan's test ($P < 0.05$). Significant effects of three-way ANOVA: *, $P <$ 0.05; **, $P < 0.01$; ***, $P < 0.001$; NS, not significant. -ABA: 0 μM ABA application; +ABA: 10 μM ABA application; AM: AM fungal inoculation; NM: non-AM fungal inoculation; Zn0: 0 mg $kg^{-1}$ Zn treatment; Zn1000: 1000 mg $kg^{-1}$ Zn treatment. AMF: AM fungal colonization; Zn: Zn stress; ABA: ABA application.

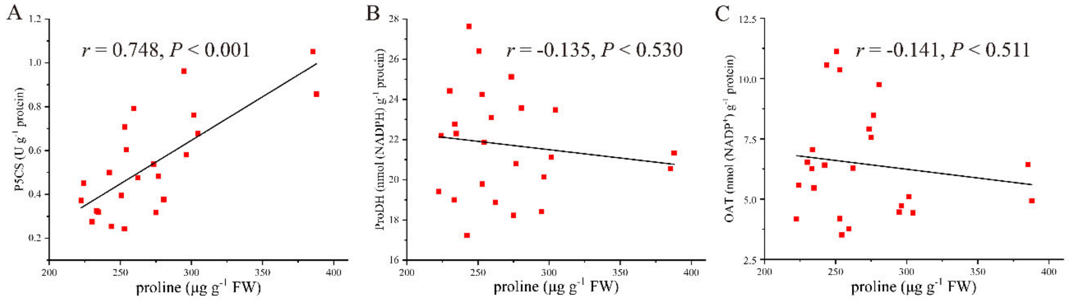


Supplementary Fig. S2 The relationships between proline content and P5CS activity, ProDH activity, OAT activity. Pearson's correlation analysis was made at the levels of 5%. $n = 24$.
