# Supplementary material for: The Synergy of Arbuscular Mycorrhizal Fungi and Exogenous Abscisic Acid Benefits Robinia pseudoacacia L. Growth through Altering the Distribution of Zn and Endogenous Abscisic Acid
Source: J Fungi (Basel). 2021 Aug 19;7(8):671. doi: 10.3390/jof7080671 (PMC8400989; doi:10.3390/jof7080671)
Supplement: Supplementary file 1 [file jof-07-00671-s001.zip › jof-1313191-supplementary/supplementary/Supplementary_Papers-from-MDPI-Staffs-Conflict-of-Interest-Statement-2021.7.20.pdf]

Multidisciplinary
Digital Publishing
Institute

MDPI AG
St. Alban-Anlage 66, 4052 Basel,
Switzerland

Tel. +41 61 683 77 34
Fax +41 61 302 89 18
www.mdpi.com

Journal of Fungi — Conflict of Interest Statement for Papers from MDPI Employees

Dear Journal of Fungi Editorial Office,

My name is Zhang Yu, I am currently working as the AE of Vaccines. I would like to submit a paper to Journal of Fungi published by MDPI. The manuscript details are as follows:

Manuscript ID: jof-1313191
Manuscript Title: The Synergy of Arbuscular Mycorrhizal Fungi and Exogenous Abscisic Acid Benefits *Robinia pseudoacacia* L. Growth through Altering the Distribution of Zn and Endogenous Abscisic Acid
Authors: Xiao Lou, Xiangyu Zhang, Yu Zhang, Ming Tang *
Corresponding Author(s): Conceptualization, Xiao Lou; Data curation, Xiao Lou; Funding acquisition, Ming Tang; Investigation, Xiao Lou and Yu Zhang; Project administration, Ming Tang; Supervision, Ming Tang; Writing – original draft, Xiao Lou; Writing – review & editing, Xiao Lou and Xiangyu Zhang.

I understand that as a MDPI employee I should declare all potential or potentially perceived conflicts of interests to the editorial office. In the manuscript, I will add a note in the Conflict of Interest part to declare that “the author xx is an employee of MDPI, however he/she does not work for the journal xx at the time of submission and publication”, if the paper can be accepted.

I understand the importance of the single blind / double blind peer-review process, even after manuscript acceptance or rejection, and confirm that neither I, nor associated parties, will check the peer review information of this paper or disclose the information to others.

Thank you very much for your consideration.

Best regards,

Zhang Yu
