# Supplementary figures and images for: The Synergy of Arbuscular Mycorrhizal Fungi and Exogenous Abscisic Acid Benefits Robinia pseudoacacia L. Growth through Altering the Distribution of Zn and Endogenous Abscisic Acid
Source: J Fungi (Basel). 2021 Aug 19;7(8):671. doi: 10.3390/jof7080671 (PMC8400989; doi:10.3390/jof7080671)

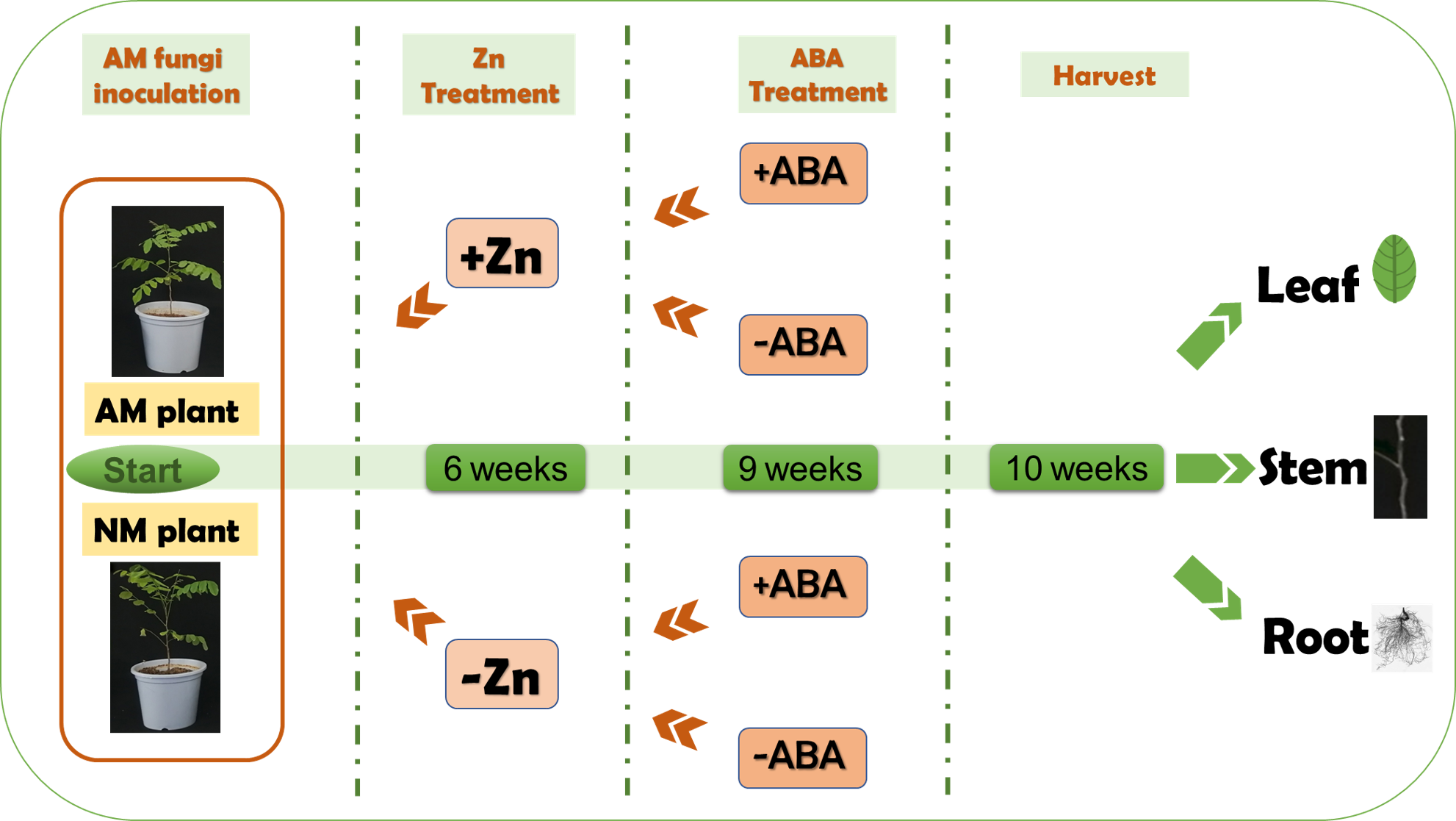

Supplement: Supplementary file 1 [file jof-07-00671-s001.zip › jof-1313191-supplementary/supplementary/Supplementary figure S1.tif]

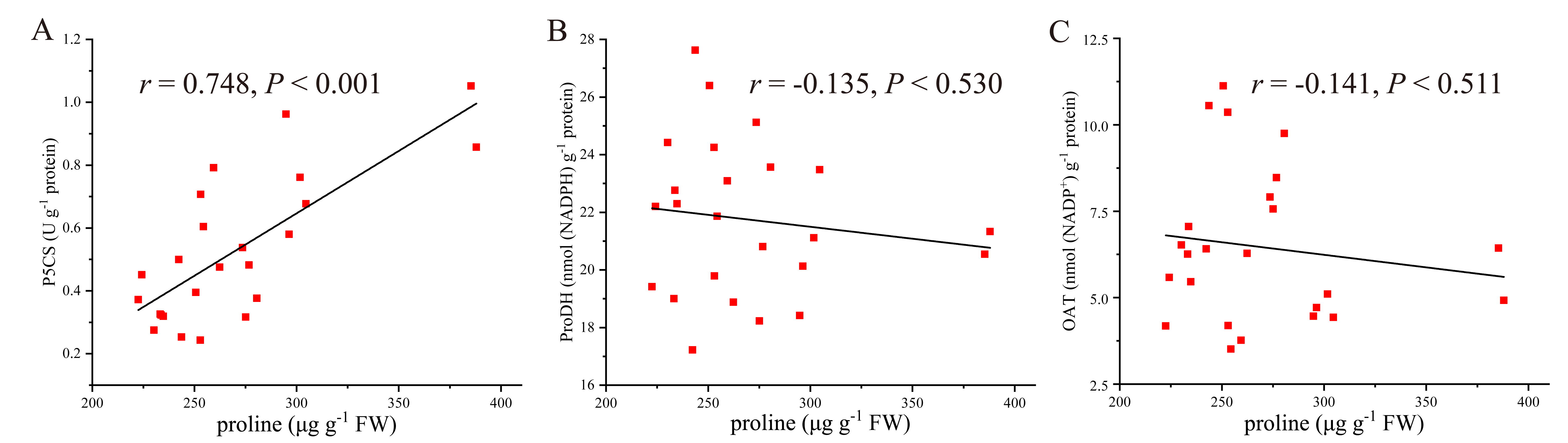

Supplement: Supplementary file 1 [file jof-07-00671-s001.zip › jof-1313191-supplementary/supplementary/Supplementary figure S2.tif]
